# Purulent Appearing Material in an Endobronchial Ultrasound-Guided Transbronchial Needle Aspiration of Mediastinal Lymph Node: A Diagnostic Challenge

**DOI:** 10.1155/2017/3851849

**Published:** 2017-10-19

**Authors:** Damaris Pena, Gilda Diaz-Fuentes, Sindhaghatta Venkatram

**Affiliations:** Division of Pulmonary and Critical Care Medicine, Bronx Lebanon Hospital Center Affiliated to Icahn School of Medicine at Mount Sinai, Bronx, NY 10457, USA

## Abstract

Endobronchial ultrasound-guided transbronchial needle aspiration (EBUS-TBNA) has increasingly been performed for the diagnosis and staging of thoracic malignancies. Findings of a necrotic lymph node raise concern for infectious process and malignancy. A hypoechoic area on ultrasound/EBUS within a lymph node without blood flow is suggestive of pathologies like infections or malignancy. Inspection of the fluid could suggest a diagnosis; clear aspirates usually suggest bronchogenic or mediastinal cysts and purulent material suggests abscesses or necrotic lymph nodes. Growing tumor cells require a blood supply; if the vascular stroma is insufficient due to rapidly growing malignant tumors this could lead to large central areas of ischemic necrosis. Necrotic aspiration of lymph nodes is not always of infectious etiology. Aspiration of fluid in EBUS-TBNA is a rare occurrence, and malignancy should be considered when purulent fluid material is obtained. We present an elderly woman who underwent bronchoscopy with EBUS-TBNA for evaluation of upper lung nodule and mediastinal lymphadenopathy. Pus-like material was obtained on needle aspiration and endobronchial biopsy and mediastinal core biopsy revealed squamous cell carcinoma.

## 1. Introduction

Ultrasound imaging has become part of the armamentarium of the pulmonologist; EBUS-TBNA plays an important role in the evaluation and diagnosis of several diseases especially malignancy [1].

EBUS-TBNA is well accepted and increasingly being used as a safe minimally invasive procedure for the diagnosis and staging of lung cancers; it carries an overall sensitivity of 89% and a negative predictive value of 91% [1–3]. It is also used to diagnose nonmalignant etiologies of enlarged mediastinal lymphadenopathy such as sarcoidosis, infections, and many rare diseases [4]. Coagulation necrosis has been described in approximately 25% of mediastinal lymphadenopathies and it is seen more often in malignancies; however, a necrotic lymph node raises concern for the presence of tuberculosis, fungal, or bacterial infections [3, 5]. We present a patient with mediastinal lymphadenopathy where EBUS-TBNA revealed fluid resembling pus and endobronchial biopsy and mediastinal core biopsy revealed squamous cell carcinoma.

## 2. Case Presentation

A 71-year-old woman from Dominican Republic was admitted for dyspnea, fever, and nonproductive cough of one-day duration. Her medical history was significant for diabetes mellitus, systolic heart failure, gastric B-cell lymphoma treated with chemotherapy, and surgically treated basal cell carcinoma of forehead. She was a heavy smoker with 40 packs/year. She denied alcohol or illicit drug use. Family history was noncontributory. There were no sick contacts, recent traveling, or occupational exposures and no history of exposure to tuberculosis.

Initial examination showed an elderly woman on respiratory distress. Chest auscultation revealed bibasilar crackles and diffuse expiratory wheezing. There were no palpable lymphadenopathy, organomegaly, or skin lesions. The rest of exam was unremarkable. Significant laboratory findings included elevated Pro-BNP; there was no leukocytosis and renal and liver function was normal. A right sided thoracentesis was performed with pleural fluid analysis revealing transudative effusion with pleural/serum LDH ratio of 0.12 and pleural/serum protein ratio of 0.19.

Patient was treated for exacerbation of heart failure with diuretics and antibiotics were given for presumptive community acquired pneumonia with clinical improvement.

**Figure 1:**
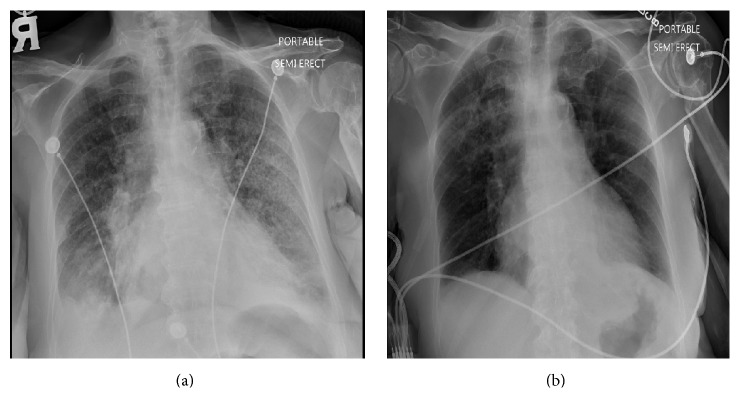
(a) CXR on admission showing bilateral small pleural effusions and infiltrates. (b) CXR 48 hours after diuresis showing resolving infiltrates.

**Figure 2:**
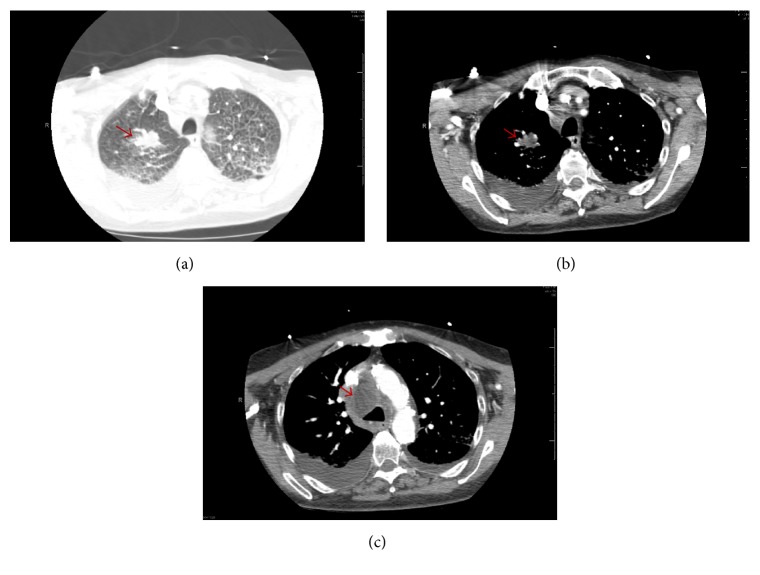
Chest CT with contrast ((a) and (b)): lung window (a) showing right upper lobe 15 mm lung nodule (red arrow), pulmonary congestion, and pleural effusion. (b) Mediastinal window showing same. (c) Mediastinal window showing hypodense right paratracheal lymph node of 3.1 cm with (red arrow).

Chest-roentgenogram (CXR) on admission showed bilateral pleural effusion and infiltrates which rapidly improved suggesting a diagnosis of heart failure rather than pneumonic process (Figure 1). A chest computed tomography (CT) revealed a 15 mm spiculated nodule in the right upper lobe, a 10 mm nodule in the left upper lobe, chronic interstitial fibrosis, and a right paratracheal lymph node measuring 3.1 cm, unchanged from a prior chest CT performed three months prior (Figure 2).

**Figure 3:**
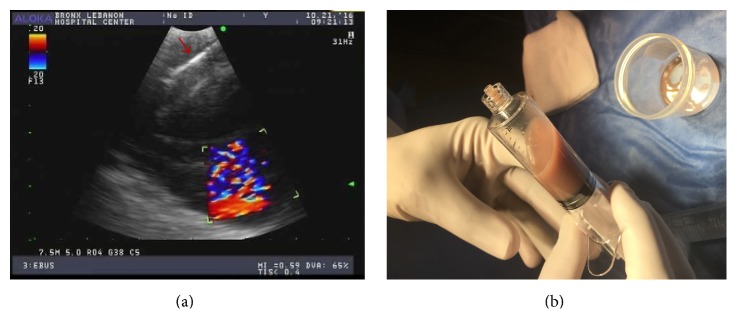
(a) EBUS needle gauge 19 inside lymph node (red arrow); no clear coagulation necrosis identified in the image. (b) Aspirated fluid.

**Figure 4:**
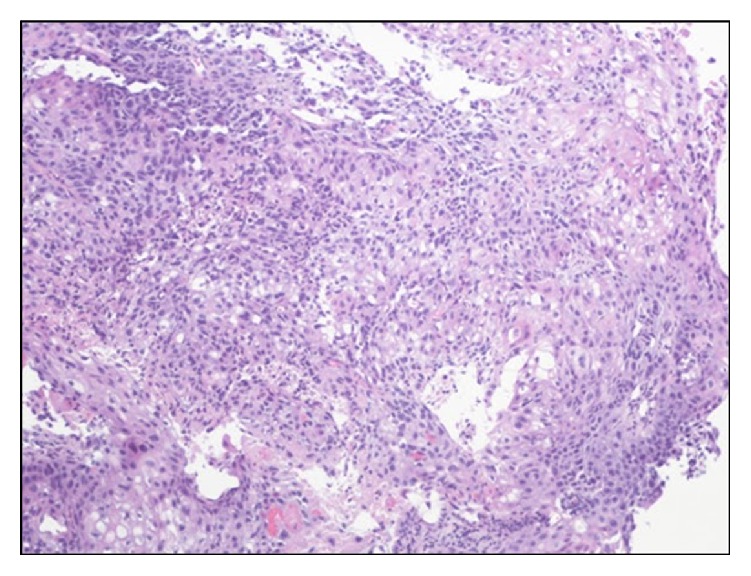
Lymph node pathology. Squamous cell carcinoma comprised sheets of malignant cells showing nuclear pleomorphism and nuclei (low magnification ×100).

Patient underwent flexible fiberoptic bronchoscopy (FFB) that revealed a small endobronchial lesion at the right upper lobe before the takeoff of anterior segmental bronchus. Endobronchial biopsy (EB) of the EBL as well as transbronchial biopsy (TBBx) of right upper nodule and EBUS-TBNA of the right paratracheal and subcarinal lymph nodes was performed. A 19′′ gauge needle was used for the EBUS-TBNA with a total of four needle passes per lymph node. Twenty ml of purulent appearing fluid was aspirated from the right paratracheal lymph node (Figure 3). Aspirated EBUS fluid showed highly atypical squamous cells in necrotic background. The EB of right upper lobe endobronchial lesion and the core biopsy of material aspirated from 4R lymph node were consistent with squamous cell carcinoma (Figure 4). Cultures of blood, urine, lung tissue, and EBUS-TBNA aspirate were all negative for bacterial infections, fungal infections, and mycobacteria. Serology for mycoplasma and urine for legionella were all negative. Collagen vascular workup was negative as well. The patient received palliative chemotherapy.

## 3. Discussion

Aspiration of liquid material in EBUS-TBNA is uncommon. A hypoechoic area on ultrasound/EBUS within lymph nodes without blood flow is suggestive of pathologies like infections or malignancy. Inspection of the fluid is suggestive of a diagnosis; clear aspirates suggest bronchogenic or mediastinal cysts and purulent material suggests infection or rarely necrotic lymph nodes. Growing tumor cells require a blood supply; if the vascular stroma is insufficient due to rapidly growing malignant tumors this could lead to large central areas of ischemic necrosis [3, 5]. Other mechanisms causing necrosis include autophagy and necroptosis. Findings of extensive necrosis have a high positive predictive value and correlate with presence of malignancy; however, similar findings have been reported with infectious process and other rare conditions such as histiocytic necrotizing lymphadenitis. Necroptosis has been documented as a line of defense against intracellular infections; it has also been implicated in a variety of disease states like ischemia, which could explain the mechanisms of necrosis in cases of malignancy like our patient [6, 7]. Accuracy, sensitivity, and specificity for ultrasound to detect necrotic malignant lymph nodes are 85%, 77%, and 93%, respectively [8]. Endobronchial ultrasound elastography has been used to better describe the stiffness of tissue during EBUS-TBNA, this is helpful in predicting benign or malignant mediastinal and hilar lymph nodes. According to the guidelines from the European federation of societies for ultrasound in medicine and biology on the use of ultrasound elastography, a sensitivity of 88% and specificity of 85% can be achieved with addition of this technique. In contrast, other studies showed a lower sensitivity (55–59%) with a similar specificity (82–85%) when compared to standard endoscopic ultrasound sampling of lymph nodes [9].

Necrotic mediastinal lymphadenopathy has been described in a wide variety of pathologies which include infectious and noninfectious etiologies. Tuberculosis is one of the most common infectious causes, especially in areas with high prevalence of tuberculosis [4]. Other infections include fungal infections, like* Histoplasma capsulatum* leading to mediastinal abscesses and necrotic mediastinal lymphadenopathies [4, 10]. Bacterial infections such as cat scratch disease, anthrax, and* Streptococcus* related mediastinitis and mediastinal abscesses have also been reported in the literature [10, 11].

Noninfectious causes include histiocytic necrotizing lymphadenitis which is a benign lymphadenitis, characterized by enlarged lymph nodes with histopathological findings of proliferation of lymphocytes and histiocytes, nuclear debris, and necrotic lesions affecting mainly young women [7]; systemic lupus erythematosus with lupus lymphadenitis can present with necrotic mediastinal lymph nodes [12]. Malignant causes of necrotic mediastinal lymphadenopathy are commonly from metastatic disease. The most frequent malignancies to metastasize to mediastinal lymph nodes include lung, esophagus, stomach, pancreas, testes, breast, and colon [4]. Coagulation necrosis has been frequently described in cervical lymph node metastasis with a prevalence of 48% in patients with head and neck squamous cell cancer. Information regarding squamous cell lung cancer presenting with necrotic mediastinal lymphadenopathies is sparse, but squamous cell carcinoma of the lung should be considered in patients with mediastinal lymph node aspirate showing liquid necrotic [8].

Lymphomas also may present as necrotic mediastinal lymphadenopathy. Usually metastases present more frequently as parenchymal lung nodules, but these are closely followed by mediastinal lymphadenopathy and pleural effusions [4].

The initial differential diagnoses in our patient were infectious etiologies versus malignancy due to the strong medical history of malignancies and imaging findings. Bronchoscopic findings were conflicting, inspection revealed an endobronchial lesion, and EBUS sampling revealed cyst-like structure and aspirated material looked purulent. It is also important to note our patient had no clinical signs to suggest a rheumatologic disorder. To our knowledge, 2 cases of EBUS-TBNA of mediastinal lymph node revealing purulent material with a final diagnosis of malignancy have been reported in the English literature [4, 5]. In both reported cases, aspiration was done in a right mediastinal node and both were squamous cell carcinoma, similar to our patient (Table 1). Although it is well known that malignancies do metastasize to mediastinal lymph nodes very frequently, the finding of purulent material is uncommon. Our report also validates sending any visible tissue on EBUS aspiration for histopathology.

## 4. Conclusion

The increased use of EBUS-TBNA to assess mediastinal diseases will likely lead to an increase in the number of patients with malignancies presenting with purulent aspirate. We suggest neoplastic conditions, especially squamous cell carcinoma, to be included in the differential diagnosis in patients where the EBUS guided needle aspiration reveals purulent material. Specimens should be analyzed to evaluate for infections like tuberculosis, fungal, and bacterial infections as well as malignancy.

## Figures and Tables

**Table 1:** Comparison of reported cases of malignancy in purulent aspirate of EBUS-TBNA.

Reference	Size of EBUS needle	Site aspirated lymph node	Final diagnosis
Madan et al. (ref. no. [4])	Not documented	Right paratracheal	Squamous cell carcinoma of the lung
Berim and Dhillon (ref. no. [5])	Not documented	Right paratracheal	Squamous cell carcinoma of the lung
Pena et al. (current case)	19 gauge	Right paratracheal	Squamous cell carcinoma of the lung

## Abbreviations

CXR: Chest-roentgenogram
CT: Computed tomography
EB: Endobronchial biopsy
EBL: Endobronchial lesion
EBUS-TBNA: Endobronchial ultrasound-guided transbronchial needle aspiration
FFB: Flexible fiberoptic bronchoscopy
Pro-BNP: Pro-B-type natriuretic peptide
TBBx: Transbronchial biopsy.
